# Tactile sensation moderates the association between hand dexterity and higher-level cognition in older adults with and without MCI

**DOI:** 10.3389/fnagi.2026.1833368

**Published:** 2026-07-03

**Authors:** Kimi Estela Kobayashi-Cuya, Ryota Sakurai, Susumu Ogawa, Hiroko Matsunaga, Keigo Hinakura, Shun Yoshikoshi, Yan Li, Maki Nishinakagawa, Hiroyuki Suzuki

**Affiliations:** 1 Research Team for Social Participation and Healthy Aging, Tokyo Metropolitan Institute for Geriatrics and Gerontology, Itabashi-ku, Tokyo, Japan; 2 Institute of Health and Sport Sciences, University of Tsukuba, Ibaraki, Japan; 3 Department of Nutrition and Dietetics, College of Nutrition, Kanto Gakuin University, Kanagawa, Japan

**Keywords:** aging, executive functioning, functional assessment, hand sensorimotor function, mild cognitive impairment, motor-cognitive integration, processing speed

## Abstract

**Background:**

Tactile sensation and hand dexterity decline with age and are associated with later-life cognition, yet it remains unclear whether tactile sensation moderates the strength of hand motor–cognitive associations. This cross-sectional study examines whether tactile sensation moderates the association between hand dexterity and higher-level cognition (executive function and processing speed) in cognitively normal (CN) older adults and those with mild cognitive impairment (MCI), and whether moderation patterns differ between these groups.

**Methods:**

Participants were 132 community-dwelling older adults aged ≥ 60 (female = 79; mean age = 73.3 ± 5.2). MCI was defined by MMSE ≥ 24 and MoCA ≤ 25. Executive function was assessed with Trail Making Test-B (TMT-B) and letter fluency (LFT); processing speed with TMT-A, Digit Symbol, and category fluency (CFT). Hand dexterity and tactile sensation were measured using the Purdue Pegboard Test and Weinstein–Semmes monofilaments. Moderation analyses tested the interaction between dexterity and tactile sensation, adjusting for demographic and health-related covariates.

**Results:**

Tactile sensation significantly moderated the association between hand dexterity and executive function and processing speed, with stronger dexterity–cognition associations among older adults with intact tactile sensation (TMT-B *p* < 0.05; LFT *p* < 0.05; TMT-A *p* < 0.01). No moderation was observed for Digit Symbol or CFT. The three-way interaction with cognitive group was not significant, indicating comparable patterns across groups. Stratified analyses showed a significant LFT interaction in the CN group (*p* < 0.05).

**Conclusion:**

Older adults with intact tactile sensation showed stronger hand dexterity–cognitive associations, highlighting sensorimotor integrity as relevant for early screening and functional evaluation. Stratified patterns suggest possible cognitive group differences that merit further study.

## Introduction

Aging involves gradual changes in both sensorimotor and cognitive systems (Murata et al., 2010; Stevens et al., 2003), which in turn can influence daily functioning and vulnerability to cognitive impairment. Among sensorimotor markers, fine motor function has emerged as a particularly sensitive indicator of cognitive vulnerability in older adults (Manelis et al., 2024), and its reliance on the integration of tactile sensory input and motor commands (Wei et al., 2024) underscores the importance of understanding how sensory and motor processes interact with cognition in later life. While age-related declines in muscle strength and practice-related factors can also affect motor performance (Seidler et al., 2010), prior work indicates that tactile sensation and fine motor dexterity are more closely linked to higher-level cognitive function, and these components therefore represent key targets for examining hand motor–cognitive integration.

Hand motor function, particularly hand dexterity, has been consistently associated with higher-level cognition, including executive function and processing speed in cognitively healthy older adults, with bidirectional longitudinal associations suggesting shared underlying mechanisms (Kobayashi-Cuya et al., 2024; Kobayashi-Cuya et al., 2018). Because dexterity relies on integrating motor planning and cognitive control, its decline may reflect not only reduced fine motor control but also broader age-related changes in neural systems involved in motor–cognitive integration (Manelis et al., 2024; Pan et al., 2025; Schneider et al., 2025). Executive function and processing speed are especially relevant to dexterous performance because they support coordination, sequencing, and efficient performance adjustment during skilled manual actions. Both domains decline early in aging with changes in frontoparietal networks that also support fine motor performance (Hedden and Gabrieli, 2004; Seidler et al., 2010).

Tactile sensation, another key component of hand sensorimotor function, also declines with age due to changes in both peripheral sensory systems and central processing pathways (Murata et al., 2010; Stevens et al., 2003). Reduced tactile sensitivity has been associated with poorer hand dexterity (Murata et al., 2010) and is more prevalent in individuals with cognitive impairment, including mild cognitive impairment (MCI) and Alzheimer’s Disease (Xu J. et al., 2024; Yang et al., 2010; Zhang et al., 2020). These patterns suggest that tactile sensation may relate to cognitive aging rather than solely peripheral hand condition. However, the sensory involvement in the association between hand dexterity and higher-level cognitive performance remains poorly understood. Most studies have examined tactile function and dexterity as independent predictors rather than interacting processes, leaving open whether tactile sensation is associated with differences in the dexterity–cognition relationship.

Although hand dexterity is associated with higher-level cognition in cognitively normal older adults, evidence for this association in MCI is limited and less consistent (Rattanavichit et al., 2022; Wu et al., 2016; Xu J. et al., 2024). Given these uncertainties, it remains unclear whether tactile sensation is associated with variation in the association between hand dexterity and higher-level cognition, underscoring the need to test whether this relationship differs by tactile-sensation status. To date, no studies have examined whether tactile sensation moderates the association between hand dexterity and higher-level cognition, or whether this moderating effect differs between cognitively healthy older adults and those with MCI. MCI is characterized by greater cognitive decline and more pronounced impairments in perceptual-motor and motor control functions (Rattanavichit et al., 2022; Wu et al., 2016). Individuals with MCI also show distinct compensation patterns, including greater prefrontal activation during sensorimotor or sensory-challenging balance tasks (Xu J. et al., 2024), which reflects increased reliance on cognitive resources to maintain sensorimotor performance. Because individuals with MCI show reduced tactile processing efficiency and greater recruitment of higher-level cognitive resources during sensorimotor tasks (Xu J. et al., 2024), tactile sensation may be more strongly related to dexterity–cognition associations in this group. Clarifying whether tactile function is associated with differences in the dexterity-cognition relationship across cognitive groups is therefore important for determining whether group-specific patterns exist in how hand sensory and motor functions interact in relation to higher-level cognition.

Therefore, the present cross-sectional study examines whether tactile sensation moderates the association between hand dexterity and higher-level cognitive functions, specifically executive function and processing speed, in community-dwelling older adults with and without MCI. By further comparing the magnitude of this moderating effect across cognitive groups, this study also aims to determine whether the strength of the dexterity-cognition association moderated by tactile sensation differs between individuals with MCI and cognitively healthy older adults.

Based on prior evidence linking tactile function to both motor and cognitive performance (Murata et al., 2010; Yang et al., 2010), we hypothesized that tactile sensation would moderate the association between dexterity and cognition, and that the magnitude of this moderating effect would be greater in older adults with MCI.

## Materials and methods

### Participants

All participants were aged 60 years and older and were drawn from two independent datasets collected during two separate recruitment periods (2020–2021 and 2024). No participant was assessed more than once. The same inclusion and exclusion criteria and testing procedures were applied across both datasets. Dataset 1 consisted of a health examination conducted at our research institute in January 2024 among community-dwelling older adults recruited from a local ward in Tokyo, and Dataset 2 consisted of baseline data collected between October 2020 and July 2021 from older adults with comparable demographic and health characteristics who had participated in a separate intervention study at the same research institution (Iizuka et al., 2025). All participants were aged 60 years and older. The datasets were merged to enhance statistical power and generalizability, yielding a combined sample of 132 participants. Because the two datasets were collected during different recruitment periods, dataset source (Dataset 1 vs. Dataset 2) was included as a fixed-effect covariate in the moderation models to adjust for potential period-related differences.

Cognitive screening was performed after data collection by trained clinical psychologists. Inclusion required a Mini-Mental State Examination (MMSE) score of ≥ 24, consistent with the established threshold for excluding dementia (Folstein et al., 1975). Based on Montreal Cognitive Assessment (MoCA) scores, 77 participants were classified as having MCI (MoCA ≤ 25), consistent with validation studies (Dong et al., 2012; Nasreddine et al., 2005) and recent literature (Islam et al., 2023), while 55 were classified as cognitively normal (CN; MoCA ≥ 26).

Exclusion criteria included a history of cerebrovascular or neurological disorders, dementia diagnosis, severe sensory or motor impairments, severe hearing or visual deficits, and mental disorders that would interfere with valid assessments. Handedness was assessed using the Edinburgh Handedness Inventory (Prichard et al., 2024). All participants scored above +40, indicating right-hand dominance; analyses were therefore conducted on the dominant hand.

This research was approved by the Institutional Review Board of Tokyo Metropolitan Institute for Gerontology and Geriatrics (Approval number R23-050, approved on August 31, 2023). All participants provided written informed consent prior to participation. This study was conducted in accordance with the Declaration of Helsinki (World Medical Association, 2013).

### Hand sensorimotor tests

#### Hand dexterity

##### Purdue Pegboard Test (PPT)

The PPT (Lafayette Instrument Company, Model 32020) was used to assess hand dexterity. This procedure was consistent with our previous application of the PPT (Kobayashi-Cuya et al., 2024). The board (19.7 × 44.9 cm) contains 25 holes in a 5 × 5 grid, and participants inserted pins one at a time for 30 s, beginning from the top of either the right or left column depending on the starting hand. The starting hand was counterbalanced, and each hand received one practice run followed by two formal trials. The mean number of correctly placed pins across the two trials was used for analysis. Dexterity scores were mean-centered prior to analysis to facilitate interpretation of interaction terms, allowing coefficients to reflect the effect of dexterity at the average level of tactile sensation and thereby enhancing clinical relevance.

#### Tactile sensation

##### Weinstein-Semmes Monofilaments

The Touch-Test Sensory Evaluator (Semmes-Weinstein Monofilaments; North Coast Medical, Inc., Morgan Hill, CA, United States) was used to assess tactile sensation. Nylon monofilaments of equal length but varying diameters were applied to seven palmar sites on each hand: the distal phalanx of the thumb, index, middle, and little fingers; the base of the proximal phalanx of the little finger; and the thenar and hypothenar regions. Each filament was pressed perpendicularly until it bent, held for approximately 2 s, and then removed. Participants responded verbally with “yes” (“hai” in Japanese) when sensation was perceived. Monofilaments sized 1.65, 2.36, 2.44, 2.83, and 3.61 were applied up to three times, while sizes 4.31 and 4.56 were applied once, with responses confirmed by correct timing.

Evaluator sizes correspond to the log10 of 10 x force (mg), expressed in grams. For example, a monofilament size of 2.83 corresponds to ∼0.07 g of force. For analysis, tactile scores were first treated as continuous values, with responses averaged across all seven sites to reflect overall tactile function. In addition, scores were dichotomized for clinical interpretation: evaluator sizes < 3.22 were classified as intact tactile sensation, while sizes ≥ 3.22 were classified as impaired tactile sensation, based on established thresholds for diminished light touch (Bell-Krotoski and Tomancik, 1987; Suda et al., 2021). The Semmes-Weinstein Monofilament Test has demonstrated good inter- and intra-rater reliability (Suda et al., 2021).

### Higher-level cognitive measures

#### Executive function tests

##### Trail Making Test Part B (TMT-B)

The TMT-B is a widely recognized measure of executive function, particularly reflecting working memory and cognitive flexibility (Arbuthnott and Frank, 2000). In this task, participants are instructed to connect sequentially ordered numbers (1–25) and the first 12 characters of the Japanese Hiragana alphabet in alternating sequence, drawing a continuous line as quickly and accurately as possible. The time required to complete the task is recorded, with shorter completion times interpreted as better executive functioning.

##### Letter Fluency Test (LFT)

The LFT is a verbal fluency task widely used as a measure of executive function, reflecting cognitive flexibility, working memory, and inhibitory control (Amunts et al., 2021). In this test, participants are instructed to generate as many words as possible beginning with a designated Japanese syllable (“ka”), within 60 s. Proper nouns and repeated words are excluded from scoring, and higher word counts indicate better executive functioning.

#### Processing speed tests

##### Trail Making Test Part A (TMT-A)

The TMT-A is widely used as an indicator of processing speed, including aspects of visual scanning, sustained attention, and motor speed (Lin et al., 2021). In this task, participants are asked to connect encircled numbers from 1 to 25 in sequential order, arranged randomly on the page. The time required to complete the sequence is recorded, with shorter completion times reflecting faster processing speed.

##### Digit symbol (WAIS-IV)

The Digit Symbol subtest of the Wechsler Adult Intelligence Scale-Fourth Edition (WAIS-IV), is a standard measure of processing speed, sustained attention, and visuomotor coordination (Joy et al., 2004). In this task, participants match symbols to digits (1–9) within 90 s, and the total number of correct pairings serves as the performance score, with higher scores reflecting higher performance.

##### Category Fluency Test (CFT)

The CFT is a widely used measure of verbal fluency that also reflects processing speed and attentional control (Gonzalez-Recober et al., 2023). In this task, participants generate as many words as possible within 60 s for the animal category. The total number of correct responses is recorded, with higher scores indicating better performance.

### Covariates

Sociodemographic characteristics including age, sex, and years of education were obtained through structured interviews. Age was modeled as a continuous variable in all analyses. Education was dichotomized into higher (≥ 13 years) and lower (≤ 12 years) categories. Functional health status was assessed using the Tokyo Metropolitan Institute of Gerontology Index of Competence (TMIG-IC). Depressive symptoms were measured using the 15-item short version of the Geriatric Depression Scale (GDS-15), with scores of ≥ 5 considered indicative of clinically relevant depressive mood, validated in Japanese populations (Sugishita et al., 2017). Comorbidities were documented as the presence or absence of self-reported conditions including hand osteoarthritis, stroke, hypertension, and diabetes. Because peripheral temperature can influence tactile sensation (Deflorio et al., 2022), hand temperature in degrees Celsius was measured on the palmar side using a digital thermometer prior to testing and entered as a covariate. In addition, cognitive group (MCI vs. CN) and dataset source (Dataset 1 vs. Dataset 2) were modeled as fixed-effect covariates.

### Statistical analysis

All analyses were conducted within a cross-sectional framework. Because participants were drawn from two independent datasets collected during different recruitment periods, dataset source (Dataset 1 vs. Dataset 2) was included as a fixed-effect covariate in all moderation models to adjust for any potential period-related differences. Age, sex, education, depressive symptoms (GDS), functional status (TMIG), cognitive group, hand temperature, and comorbidities (hand osteoarthritis, stroke, hypertension, and diabetes) were also included as covariates. Group differences between CN and MCI participants (Table 1) were examined using independent-samples t-tests and Chi-square tests for continuous and categorical variables, respectively. Mann-Whitney U tests were applied to log-transformed values when distributions were non-normal

**TABLE 1:** Descriptives statistics by cognitive group.

Variable	CN (*n* = 55)	MCI (*n* = 77)	*p*-value
Age, mean (SD)	72.2 (5.4)	74.0 (5.1)	0.057
Female, n (%)	38 (69.1)	41 (53.2)	0.074^a^
Education level (≥ 13y), n (%)	35 (63.6)	49 (63.6)	1.000^a^
GDS (≥ 5), n (%)	17 (32.1)	21 (28.4)	0.640^a^
TMIG-IC, mean (SD)	11.7 (1.2)	11.8 (1.4)	0.638
Comorbidities			
Hand osteoarthritis, n (%)	0 (0)	5 (6.8)	0.071^a^
Stroke, n (%)	0 0)	2 (2.7)	0.507^a^
Hand sensorimotor variables			
Hand dexterity (PPT), mean (SD)	14.2 (1.7)	13.4 (1.9)	**0.017**
Handgrip Strength (Kg), mean (SD)	27.3 (7.8)	27.4 (7.0)	**0.898**
Tactile impairment (≥ 3.22), n (%)	18 (32.7)	32 (41.6)	0.364a
Cognitive variables			
Executive function			
TMT-B, mean (SD)	98.5 (37.3)	128.9 (55.6)	**0.002^b^**
LFT, mean (SD)	12.2 (4.0)	10.4 (3.5)	**0.008**
Processing speed			
TMT-A, mean (SD)	45.1 (21.0)	47.7 (17.4)	0.173^b^
CFT, mean (SD)	16.9 (3.5)	16.1 (4.5)	0.219
Digit symbol, mean (SD)	70.8 (15.6)	62.1 (14.7)	**0.001**

CN, Cognitively normal; MCI, mild cognitive impairment; GDS, Geriatric Depression Scale; TMIG-IC, Tokyo Metropolitan Institute of Gerontology-Index of Competence; PPT, Purdue Pegboard Test (number of pegs); TMT, Trail Making Test; LFT, Letter Fluency Test; CFT, Category Fluency Test.

^a^Chi-square test was performed.

^b^Mann-Whitney U test was performed with log- transformed values. Bold *p*-values indicate statistical significance (*p* < 0.05).

Linear mixed-effects models (LMMs) were used to examine whether tactile sensation moderated the association between hand dexterity and higher-level cognitive performance. LMMs were selected because they flexibly accommodate fixed-effect predictors and provide robust estimation under missing-at-random assumptions (Brown and Prescott, 2014). A random intercept for participants was included to account for individual-level variability. Because the study design was cross-sectional and each participant contributed a single observation, no random slopes were specified. Group-level covariates such as cognitive status and dataset source were modeled as fixed effects, consistent with the cross-sectional design. Estimation via maximum likelihood is robust to missing data under the assumption of missing at random, thereby reducing potential bias compared with complete-case linear regression (Brown, 2021).

The dependent variables consisted of measures of executive function (Trail Making Test Part B, log-TMT-B; Letter Fluency Test, LFT) and processing speed (Trail Making Test Part A, log-TMT-A; Digit Symbol Substitution Test, DSST; Category Fluency Test, CFT). Each cognitive test was analyzed separately to preserve the distinct cognitive demands of each measure. Because TMT-A and TMT-B scores exhibited skewed distributions, log transformation (log-TMT-A, log-TMT-B) was applied to improve normality prior to analysis.

The primary predictors were hand dexterity (continuous, centered) and tactile sensation (binary: intact vs. impaired), entered as main effects and as an interaction term (dexterity × tactile sensation) to test moderation. Moderation was evaluated through the interaction term, consistent with the definition that the effect of one predictor on an outcome is conditional on the level of another variable (Hayes, 2022). A three-way interaction (dexterity × tactile sensation × cognitive group) was additionally specified to evaluate whether moderation effects differed between CN and MCI participants. Stratified analyses were conducted within each group to compare interaction coefficients.

All models included covariates entered as fixed effects: age, sex, education, depressive symptoms (GDS), functional status (TMIG), cognitive group, dataset source, hand temperature, and comorbidities (hand osteoarthritis, stroke, hypertension, and diabetes). Statistical significance was set at *p* < 0.05. Analyses were conducted using IBM SPSS Statistics, version 23 (IBM Corp, Armonk, NY, United States), with linear mixed-effects models estimated via maximum likelihood.

## Results

### Descriptive statistics

Table 1 presents demographic, hand sensorimotor, and cognitive characteristics by cognitive group. As expected, older adults with MCI showed lower performance on hand dexterity (*p* < 0.05), TMT-B, LFT, and Digit Symbol (all *p* < 0.01), consistent with established patterns of reduced motor and cognitive performance in the context of cognitive impairment. Tactile sensation did not differ significantly between groups. All moderation models were adjusted for age, sex, education, depressive symptoms, functional status, comorbidities, and dataset source. In the full-sample models (Table 2), cognitive group was also included as a covariate, whereas in the stratified models (Table 3), cognitive group was inherent to the stratification.

**TABLE 2:** Moderation effects of tactile sensation on the association between hand dexterity and higher-level cognitive outcomes.

Outcome	Dexterity (PPT)^a^ β (95%CI)	Tactile β (95%CI)	Interaction β (95%CI)	Interaction (*p*-value)
Executive function				
TMT-B^b^	6.96 (–2.90,16.25)	–17.41 (–34.82, –0.35)	–11.26 (–22.05, –0.46)	**0.041**
LFT	–0.51 (–1.29, 0.27)	0.03 (–1.45, 1.51)	0.98 (0.10, 1.87)	**0.029**
Processing speed				
TMT-A^b^	2.47 (–0.98, 5.92)	–6.02 (–12.59, 0.56)	–6.53 (–10.54, –2.71)	**0.001**
CFT	–0.11(–0.98, 0.75)	–0.32 (–1.96, 1.31)	0.17 (–0.81, 1.16)	0.727
Digit Symbol	0.24 (-2.88, 3.36)	1.67 (–4.24, 7.57)	3.00 (–0.53, 6.54)	0.095

PPT, Purdue Pegboard Test (number of pegs); TMT, Trail Making Test; LFT, Letter Fluency Test; CFT, Category Fluency Test. Each model included main effects and the dexterity × tactile sensation interaction, and was adjusted for cognitive group, age, sex, education, GDS, TMIG-IC, comorbidities, and data source. Tactile sensation was coded as intact vs. impaired.

^a^Dexterity was mean-centered prior to analysis to facilitate interpretation of interaction terms.

^b^Values are back-transformed from the natural log scale to seconds for interpretability. Bold *p*-values indicate statistical significance (*p* < 0.05).

**TABLE 3:** Moderation effects of tactile sensation on the association between hand dexterity and higher-level cognitive outcomes, stratified by cognitive group.

Group	Outcome	Dexterity (PPT)^a^ β (95%CI)	Tactile β (95%CI)	Interaction β (95%CI)	Interaction (*p*-value)
Executive function					
**CN**	TMT-B^b^	–0.98 (–15.76, 13.79)	–21.67 (–44.32, 0.98)	–1.97 (–17.73, 12.80)	0.744
	LFT	–1.59 (–3.35, 0.16)	–0.33 (–3.08, 2.42)	1.89 (0.05, 3.73)	**0.045**
MCI	TMT-B^b^	7.74 (–7.74, 23.22)	–9.48 (–21.66, 27.07)	–7.22 (–15.34, 1.08)	0.141
	LFT	–0.29 (–1.21, 0.62)	–0.34 (–2.42, 1.73)	0.85 (–0.27, 1.97)	0.133
Processing speed					
CN	TMT-A^b^	0.90 (–7.22, 8.57)	–9.48 (–21.66, 27.07)	–7.22 (–15.34, 1.08)	0.087
	CFT	–0.19 (–1.64, 1.26)	–0.67 (-2.95, 1.60)	–0.19 (–1.64, 1.26)	0.788
	Digit symbol	0.64 (–6.24, 7.52)	3.59 (–7.19, 14.38)	4.52 (–2.69, 11.73)	0.212
MCI	TMT-A^b^	3.34 (–9.07, 7.16)	–0.95 (–10.02, 8.11)	–4.58 (–9.55, 0.38)	0.070
	CFT	–1.81 (–1.41, 1.05)	0.47 (–2.29, 3.24)	–0.15 (–1.64, 1.35)	0.845
	Digit symbol	0.63 (–3.31, 4.56)	–0.80 (–9.68, 8.08)	0.94 (–3.83, 5.71)	0.694

PPT, Purdue Pegboard Test (number of pegs); CN, Cognitively normal; MCI, Mild cognitive impairment; TMT, Trail Making Test; LFT, Letter Fluency Test; CFT, Category Fluency Test. Each model included main effects and the dexterity × tactile sensation interaction, and was adjusted for age, sex, education, GDS, TMIG-IC, comorbidities, and data source. Tactile sensation was coded as intact vs. impaired.

^a^Dexterity was mean-centered prior to analysis to facilitate interpretation of interaction terms.

^b^Values are back-transformed from the natural log scale to seconds for interpretability. Bold *p*-values indicate statistical significance (*p* < 0.05).

### Two-way interaction between hand dexterity and tactile sensation

**FIGURE 1:**
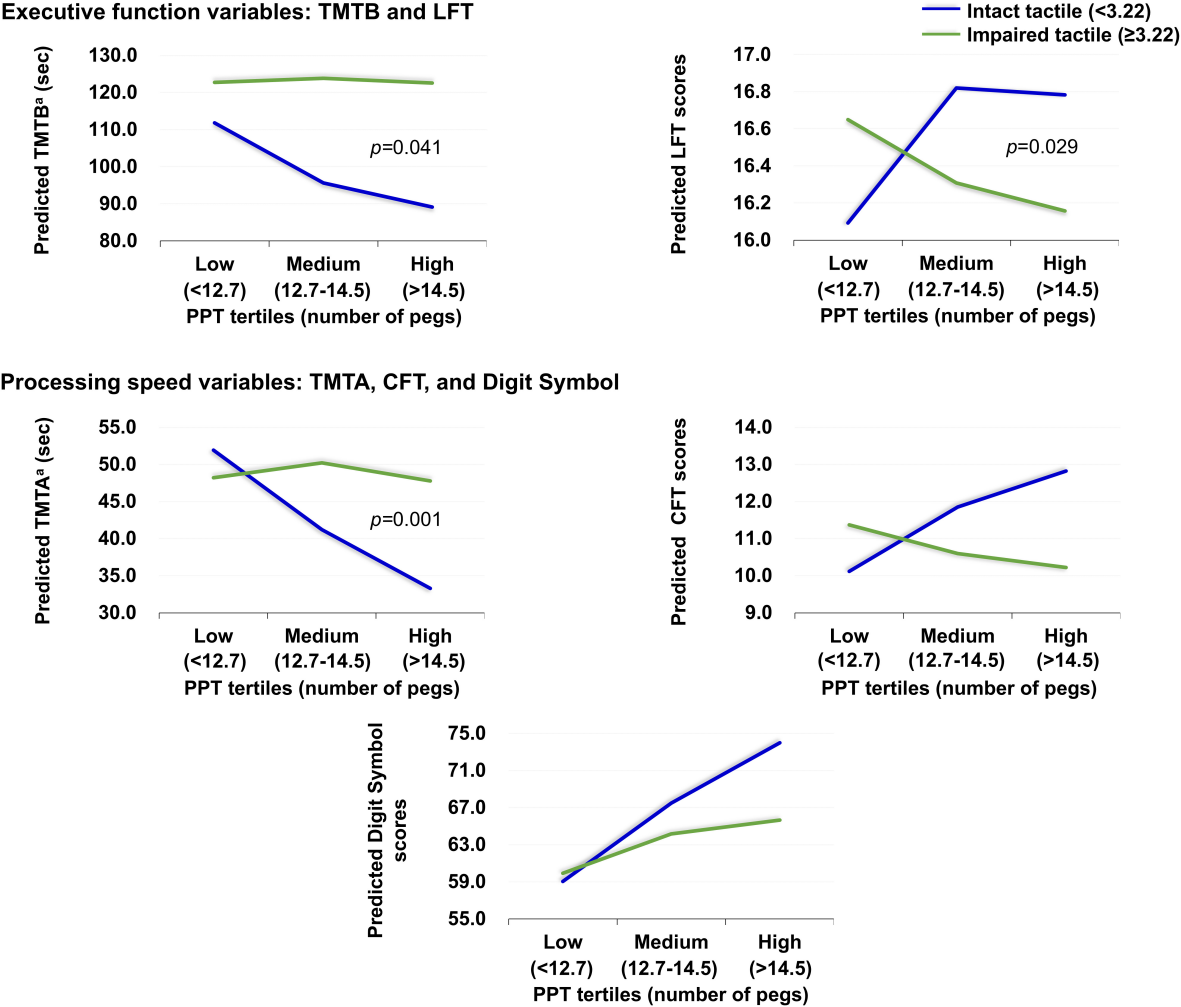
Moderation effects of tactile sensation on the association between hand dexterity and higher-level cognitive outcomes across PPT tertiles. Predicted scores for executive function (TMT-B, LFT) and processing speed (TMT-A, CFT, Digit Symbol) are plotted across PPT tertiles. Tactile sensation was stratified as intact (< 3.22) or impaired (≥ 3.22). PPT, Purdue Pegboard Test (number of pegs); TMT, Trail Making Test; LFT, Letter Fluency Test; CFT, Category Fluency Test. *^a^*TMT-A and TMT-B values are back-transformed from the natural log scale to seconds for interpretability.

Results from linear mixed-effects models are presented in Table 2. Significant two-way interaction effects between hand dexterity and tactile sensation were observed for TMT-B (β = –11.26, *p* < 0.05), LFT (β = 0.98, *p* < 0.05), and TMT-A (β = –6.53, *p* < 0.01), indicating that tactile sensation moderated associations between dexterity and both executive function and processing speed. No significant moderation effects were found for CFT or Digit Symbol. As illustrated in Figure 1, slopes were steeper among older adults with intact tactile sensation (monofilament size < 3.22; target force < 0.16 g), reflecting stronger dexterity–cognition associations at higher tactile sensitivity levels.

### Three-way interaction with cognitive group

The three-way interaction among hand dexterity, tactile sensation, and cognitive group was not statistically significant (Supplementary Table S1), suggesting that the magnitude of the moderating effects did not differ reliably between CN and MCI participants.

### Stratified analysis by cognitive groups

Table 3 presents stratified models. In the CN group, tactile sensation significantly moderated the association between hand dexterity and LFT (β = 1.89, *p* < 0.05). In the MCI group, the interaction term for TMT-B showed a larger coefficient than in CN but did not reach statistical significance. These patterns suggest potential variability in moderation effects across cognitive profiles; however, interpretations should remain cautious given the uncertainty in the estimates.

## Discussion

This study is the first to examine whether tactile sensation moderates the association between hand dexterity and higher-level cognitive performance in community-dwelling older adults with and without MCI. Across the full sample, tactile sensation significantly moderated the relationship between dexterity and executive function (TMT-B, LFT) and processing speed (TMT-A). Although the three-way interaction with cognitive group was not significant, stratified analyses suggested domain-specific patterns, including a significant moderation effect for LFT in cognitively intact older adults and larger—but nonsignificant—coefficients for TMT-B in individuals with MCI. These findings suggest that tactile sensation is associated with individual differences in the dexterity–cognition relationship, potentially reflecting hand-related sensorimotor processes relevant for higher-level cognitive performance among older adults.

### Tactile sensation as a contributor to hand dexterity–cognitive integration

The observed moderation indicates that tactile sensation is associated with variability in how tactile sensory information relates to higher-level cognitive processes involved in dexterous actions, rather than determining dexterity or cognitive performance levels. That is, individuals with impaired tactile sensation may still demonstrate high dexterity or low executive function or processing speed, reflecting the heterogeneity of sensorimotor and higher-level cognitive function in aging. Importantly, tactile sensation appears to condition the strength of the dexterity–cognition association, indicating that sensory input is one contributor to variability in how motor and cognitive processes interact during dexterous actions.

Tactile feedback plays a role in the precision and regulation of fine motor control (e.g., adjusting grip, finger movement coordination, error detection), and prior work in more homogeneous samples has reported associations between tactile sensitivity and manual dexterity (Murata et al., 2010). In the present study, this association was small and non-significant, which is likely attributable to the broader heterogeneity of our cohort. This pattern suggests that tactile sensation may be more relevant for how sensory information is incorporated during cognitively supported fine-motor actions, rather than directly reflecting overall dexterity levels. Within this framework, older adults with more intact tactile sensation may be better able to utilize tactile cues during motor planning and monitoring, potentially reducing the cognitive resources required to support dexterous performance. This interpretation aligns with evidence that tactile discrimination becomes more cognitively demanding when tactile input is degraded (Xu J. et al., 2024). Thus, tactile sensation may play a meaningful role in how sensory information is integrated with motor control processes during dexterous actions, providing a plausible pathway through which individual differences in tactile function could shape motor–cognitive interactions.

**FIGURE 2:**
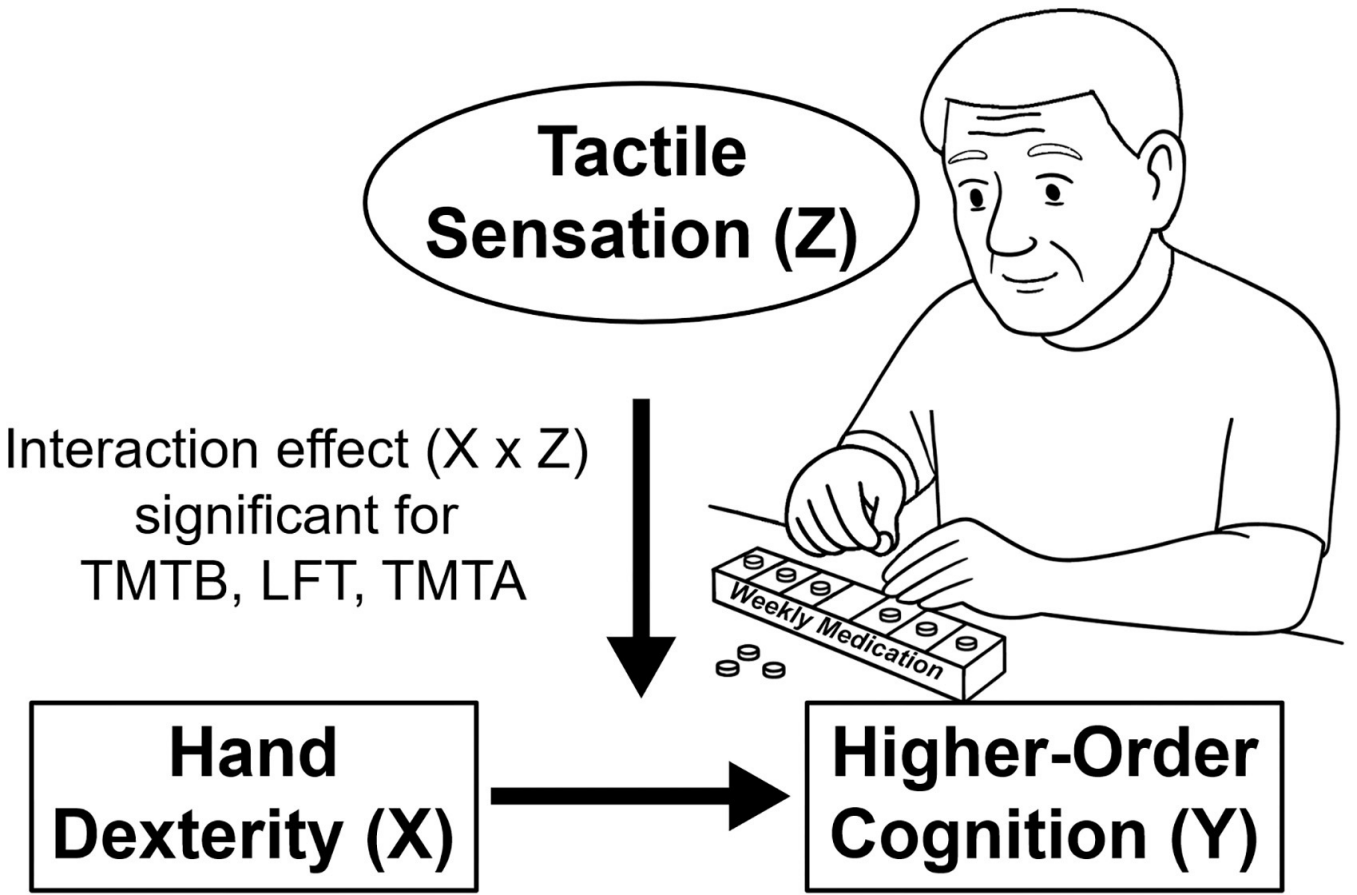
Conceptual model illustrating the moderating role of tactile sensation (Z) in the association between hand dexterity (X) and higher-level cognition (Y) in older adults. Significant X ×Z interactions were observed for TMT-B, LFT, and TMT-A.

Notably, the moderation effects observed for executive function measures such as TMT-B and LFT, which rely on cognitive flexibility, shifting, working memory, and inhibitory control (Amunts et al., 2021; Arbuthnott and Frank, 2000), suggest that dexterous actions requiring careful control and flexible adjustment may be particularly sensitive to the quality of tactile feedback, which helps support the coordination between sensory feedback and executive control during fine-motor performance. These executive processes support monitoring, error correction, and adaptive adjustments during fine motor actions (Diamond, 2013; Seidler et al., 2010), and reduced tactile input may increase reliance on these cognitive resources to maintain dexterous performance. This interpretation aligns with aging theories proposing greater cognitive engagement when sensory feedback becomes less efficient (Li et al., 2001; Seidler et al., 2010) and is consistent with our finding that hand dexterity was related to higher-level cognition only among individuals with intact tactile sensation. These mechanisms may also explain why executive functions are important for instrumental activities of daily living (IADLs) such as meal preparation, shopping, and medication management (Downey et al., 2025), as represented in the conceptual schematic in Figure 2.

A similar pattern was observed for TMT-A, which reflects psychomotor processing speed, visuomotor integration, and continuous motor control (Lin et al., 2021), suggesting that tactile sensation may support efficient perceptual-motor coordination during basic visuomotor tasks. These tasks are foundational for both basic ADLs (BADLs; e.g., buttoning, grooming) and IADLs (Royall et al., 2004). In contrast, no moderation effects of hand sensation were observed for Digit Symbol or CFT. Digit Symbol performance relies primarily on symbol–digit mapping and sustained attention, whereas category fluency depends more heavily on semantic retrieval processes, which may be less dependent on sensorimotor or tactile-guided motor control. Thus, tactile sensation may be most relevant for cognitive tasks that share overlapping sensorimotor demands or require rapid integration of sensory feedback with higher-level control processes, such as executive functioning or visuomotor processing.

### Cognitive group differences: exploratory interpretation of stratified patterns

Although the three-way interaction with cognitive group was not significant, exploratory stratified analyses revealed suggestive patterns that, consistent with descriptive subgroup guidance (Gelman and Hill, 2006), warrant cautious interpretation. Among cognitively normal older adults, tactile sensation significantly moderated the association between hand dexterity and LFT, suggesting that intact tactile input may be relevant for the cognitive demands involved in dexterous actions in healthy aging. In contrast, individuals with MCI showed a larger—but nonsignificant—interaction coefficient for TMT-B (β = –7.22 vs. –1.97 in CN), a pattern that may reflect greater variability in sensorimotor–cognitive relationships within this group. This uncertainty limits statistical power and hinders the ability to draw reliable conclusions about group differences.

### Clinical and practical implications

These findings suggest that tactile sensation is relevant to how hand dexterity relates to higher-level cognitive performance in later life. Because the dexterity–cognition association was strongest among individuals with intact tactile sensation, the results indicate that sensory and motor factors are both important for understanding how older adults integrate tactile feedback with cognitive control during everyday tasks. Considering both tactile sensation and dexterity, rather than either hand domain alone, may therefore be useful when evaluating functional vulnerability or identifying individuals who could benefit from further assessment or intervention.

Because both tactile sensation and hand dexterity decline with age, incorporating brief tactile assessments such as monofilament testing into routine evaluations may offer a feasible approach for identifying older adults whose sensorimotor changes may place greater demands on cognitive resources during everyday tasks. Because tactile sensation is not typically included in routine community health assessments, these findings highlight a potential gap in current screening practices. Integrating simple tactile measures alongside dexterity tests could help detect subtle functional vulnerability earlier, particularly for individuals who rely more heavily on cognitive control when sensory feedback is reduced. This multidomain approach may help clarify whether dexterity performance reflects sensory limitations, cognitive demands, or their interaction, supporting more informed decisions about who may benefit from additional evaluation or support in both clinical and community settings.

Although causal pathways cannot be inferred from this cross-sectional design, the observed moderation patterns suggest that maintaining or enhancing tactile function may be relevant for supporting efficient motor–cognitive integration. Activities that engage tactile discrimination, fine-motor coordination, or perceptual–motor integration may help promote the use of sensory feedback during dexterous actions, potentially reducing cognitive load and supporting functional independence. These results underscore the value of incorporating tactile sensory evaluation into assessments of motor and cognitive aging and point to hand sensation as a promising target for future longitudinal and intervention studies aimed at mitigating age-related declines in motor–cognitive efficiency.

### Strengths and limitations

This study has several strengths. It is the first to examine whether tactile sensation moderates the association between hand dexterity and higher-level cognition in community-dwelling older adults, extending prior work that has typically considered these domains separately. Combining two datasets increased sample diversity and statistical power, and adjusting for established covariates relevant to dexterity–cognition associations reduced potential confounding. The inclusion of both cognitively normal individuals and those with MCI allowed for exploratory consideration of potential group differences, and the use of well-validated measures of tactile sensation, dexterity, and higher-level cognition strengthens the interpretability of the findings. The focus on community-dwelling older adults also enhances ecological relevance, particularly given that tactile sensation is rarely assessed in routine community health evaluations.

Several limitations should also be acknowledged. The cross-sectional design precludes conclusions about directionality or causality, and longitudinal studies are needed to determine whether changes in tactile function are associated with hand motor–cognitive trajectories over time. The small subgroup sample sizes limited power to detect three-way interactions; therefore, subgroup patterns should be interpreted as exploratory. Tactile function was assessed using monofilaments, which capture threshold-level sensitivity but do not encompass other aspects of somatosensory function such as proprioception, vibration sense, or tactile discrimination. Similarly, dexterity was represented by the dominant-hand task in this analysis, and future work incorporating additional dexterity measures may provide a more comprehensive understanding of sensorimotor–cognitive relationships. Finally, although participants were community-dwelling older adults, the sample may not fully represent the broader aging population, and replication in more diverse cohorts is warranted.

## Conclusion

This study demonstrates that tactile sensation significantly moderates the association between hand dexterity and both executive function and processing speed in community-dwelling older adults. Specifically, hand dexterity was related to executive function (TMT-B, LFT) and processing speed (TMT-A) only among individuals with intact tactile sensation, indicating that the strength of the dexterity-cognition relationship varies depending on tactile function. Although subgroup analyses did not reveal statistically reliable differences between cognitively normal individuals and those with MCI, the observed patterns highlight the need for further investigation into potential variability in sensorimotor–cognitive coupling across cognitive profiles. These findings highlight tactile sensation as an underrecognized yet clinically relevant component of hand motor–cognitive functioning. Incorporating brief tactile assessments into routine evaluations may help contextualize dexterity performance, particularly when interpreting its relationship with higher-level cognitive function. Longitudinal and intervention studies are needed to clarify causal pathways and to determine whether maintaining or enhancing tactile function is associated with more efficient hand motor–cognitive integration and support independence in daily life among older adults.

## Data Availability

The raw data supporting the conclusions of this article will be made available by the authors, without undue reservation.

## References

[B1] Amunts J. Camilleri J. A. Eickhoff S. B. Patil K. R. Heim S. von Polier G. G. et al. (2021). Comprehensive verbal fluency features predict executive function performance. *Sci. Rep.* 11:6929. 10.1038/s41598-021-85981-1 33767208 PMC7994566

[B2] Arbuthnott K. Frank J. (2000). Trail making test, part B as a measure of executive control: Validation using a set-switching paradigm. *J. Clin. Exp. Neuropsychol.* 22 518–528. 10.1076/1380-3395(200008)22:4;1-0;ft518 10923061

[B3] Bell-Krotoski J. Tomancik E. (1987). The repeatability of testing with Semmes-Weinstein monofilaments. *J. Hand Surg. Am.* 12 155–161. 10.1016/s0363-5023(87)80189-2 3805636

[B4] Brown H. Prescott R. (2014). *Applied Mixed Models in Medicine.* England: Wiley.

[B5] Brown V. A. (2021). An introduction to linear mixed-effects modeling in R. *Adv. Methods Pract. Psychol. Sci.* 4:2515245920960351. 10.1177/2515245920960351

[B6] Deflorio D. Di Luca M. Wing A. M. (2022). Skin and mechanoreceptor contribution to tactile input for perception: A review of simulation models. *Front. Hum. Neurosci.* 16:862344. 10.3389/fnhum.2022.862344 35721353 PMC9201416

[B7] Diamond A. (2013). Executive functions. *Annu. Rev. Psychol.* 64 135–168. 10.1146/annurev-psych-113011-143750 23020641 PMC4084861

[B8] Dong Y. Lee W. Y. Basri N. A. Collinson S. L. Merchant R. A. Venketasubramanian N. et al. (2012). The montreal cognitive assessment is superior to the mini-mental state examination in detecting patients at higher risk of dementia. *Int. Psychogeriatr.* 24 1749–1755. 10.1017/s1041610212001068 22687278

[B9] Downey N. Daly B. Dukelow T. Curtin D. Hartigan I. (2025). Performance-Based measures of executive function through instrumental activities of daily living: Systematic review for early detection of mild cognitive impairment. *Age Ageing* 54:afaf318.076. 10.1093/ageing/afaf318.076

[B10] Folstein M. F. Folstein S. E. McHugh P. R. (1975). Mini-mental state”. A practical method for grading the cognitive state of patients for the clinician. *J. Psychiatr. Res.* 12 189–198. 10.1016/0022-3956(75)90026-6 1202204

[B11] Gelman A. Hill J. (2006). *Data Analysis Using Regression and Multilevel/Hierarchical Models.* Cambridge: Cambridge University Press.

[B12] Gonzalez-Recober C. Nevler N. Shellikeri S. Cousins K. A. Q. Rhodes E. Liberman M. et al. (2023). Comparison of category and letter fluency tasks through automated analysis. *Front. Psychol.* 14:1212793. 10.3389/fpsyg.2023.1212793 37901072 PMC10600440

[B13] Hayes A. F. (2022). *Introduction to Mediation, Moderation, and Conditional Process Analysis: A Regression-based Approach.* New York, NY: Guilford Press.

[B14] Hedden T. Gabrieli J. D. E. (2004). Insights into the ageing mind: A view from cognitive neuroscience. *Nat. Rev. Neurosci.* 5 87–96. 10.1038/nrn1323 14735112

[B15] Iizuka A. Ogawa S. Cho D. Fujihira K. Li Y. Sato K. et al. (2025). The effects of the MONOZUKURI program on executive function among community-dwelling older adults: A randomized controlled trial. *Brain Behav.* 15:e70888. 10.1002/brb3.70888 41014238 PMC12475996

[B16] Islam N. Hashem R. Gad M. Brown A. Levis B. Renoux C. et al. (2023). Accuracy of the montreal cognitive assessment tool for detecting mild cognitive impairment: A systematic review and meta-analysis. *Alzheimers Dement.* 19 3235–3243. 10.1002/alz.13040 36934438

[B17] Joy S. Kaplan E. Fein D. (2004). Speed and memory in the WAIS-III digit symbol–coding subtest across the adult lifespan. *Arch. Clin. Neuropsychol.* 19 759–767. 10.1016/j.acn.2003.09.009 15288329

[B18] Kobayashi-Cuya K. E. Sakurai R. Sakuma N. Suzuki H. Ogawa S. Takebayashi T. et al. (2024). Bidirectional associations of high-level cognitive domains with hand motor function and gait speed in high-functioning older adults: A 7-year study. *Arch. Gerontol. Geriatr.* 117:105232. 10.1016/j.archger.2023.105232 37956584

[B19] Kobayashi-Cuya K. E. Sakurai R. Sakuma N. Suzuki H. Yasunaga M. Ogawa S. et al. (2018). Hand dexterity, not handgrip strength, is associated with executive function in Japanese community-dwelling older adults: A cross-sectional study. *BMC Geriatr.* 18:192. 10.1186/s12877-018-0880-6 30143006 PMC6109297

[B20] Li K. Z. Lindenberger U. Freund A. M. Baltes P. B. (2001). Walking while memorizing: Age-related differences in compensatory behavior. *Psychol. Sci.* 12 230–237. 10.1111/1467-9280.00341 11437306

[B21] Lin Z. Tam F. Churchill N. W. Lin F. H. MacIntosh B. J. Schweizer T. A. et al. (2021). Trail making test performance using a touch-sensitive tablet: Behavioral kinematics and electroencephalography. *Front. Hum. Neurosci.* 15:663463. 10.3389/fnhum.2021.663463 34276323 PMC8281242

[B22] Manelis A. Hu H. Satz S. (2024). The relationship between reduced hand dexterity and brain structure abnormality in older adults. *Geriatrics* 9:165. 10.3390/geriatrics9060165 39727824 PMC11728121

[B23] Murata J. Murata S. Hiroshige J. Ohtao H. Horie J. Kai Y. (2010). The influence of age-related changes in tactile sensibility and muscular strength on hand function in older adult females. *Intern. J. Gerontol.* 4 180–183. 10.1016/j.ijge.2010.11.004

[B24] Nasreddine Z. S. Phillips N. A. Bédirian V. Charbonneau S. Whitehead V. Collin I. et al. (2005). The montreal cognitive assessment, MoCA: A brief screening tool for mild cognitive impairment. *J. Am. Geriatr. Soc.* 53 695–699. 10.1111/j.1532-5415.2005.53221.x 15817019

[B25] Pan D. Wei D. Zhao Y. Zhang J. Zhao Y. Shen J. et al. (2025). From fingers to brain: Virtual reality-based test capturing fine hand movements predicts cognitive function in older adults. *Innov. Aging* 9:igaf062. 10.1093/geroni/igaf062 40979467 PMC12448613

[B26] Prichard E. C. Clarkson E. M. Christman S. D. (2024). Differences between consistent and inconsistent handedness remain consistently interesting: Ten years of research on the consistency of handedness with the edinburgh handedness inventory. *Percept. Mot. Skills* 131 5–16. 10.1177/00315125231217624 37994625

[B27] Rattanavichit Y. Chaikeeree N. Boonsinsukh R. Kitiyanant K. (2022). The age differences and effect of mild cognitive impairment on perceptual-motor and executive functions. *Front. Psychol.* 13:906898. 10.3389/fpsyg.2022.906898 35967690 PMC9366843

[B28] Royall D. R. Palmer R. Chiodo L. K. Polk M. J. (2004). Declining executive control in normal aging predicts change in functional status: The freedom house study. *J. Am. Geriatr. Soc.* 52 346–352. 10.1111/j.1532-5415.2004.52104.x 14962147

[B29] Schneider T. R. Felbecker A. von Mitzlaff B. Weissofner G. Meier S. Eggenberger P. et al. (2025). Hand dexterity and mobility independently predict cognition in older adults: A multi-domain regression analysis. *Front. Aging Neurosci.* 17:1624307. 10.3389/fnagi.2025.1624307 40933827 PMC12417501

[B30] Seidler R. D. Bernard J. A. Burutolu T. B. Fling B. W. Gordon M. T. Gwin J. T. et al. (2010). Motor control and aging: Links to age-related brain structural, functional, and biochemical effects. *Neurosci. Biobehav. Rev.* 34 721–733. 10.1016/j.neubiorev.2009.10.005 19850077 PMC2838968

[B31] Stevens J. C. Alvarez-Reeves M. Dipietro L. Mack G. W. Green B. G. (2003). Decline of tactile acuity in aging: A study of body site, blood flow, and lifetime habits of smoking and physical activity. *Somatosens. Mot. Res.* 20 271–279. 10.1080/08990220310001622997 14675966

[B32] Suda M. Kawakami M. Okuyama K. Ishii R. Oshima O. Hijikata N. et al. (2021). Validity and reliability of the semmes-weinstein monofilament test and the thumb localizing test in patients with stroke. *Front. Neurol.* 11:625917. 10.3389/fneur.2020.625917 33584520 PMC7873561

[B33] Sugishita K. Sugishita M. Hemmi I. Asada T. Tanigawa T. (2017). A validity and reliability study of the japanese version of the geriatric depression scale 15 (GDS-15-J). *Clin. Gerontol.* 40 233–240. 10.1080/07317115.2016.1199452 28452641

[B34] Wei Y. Marshall A. G. McGlone F. P. Makdani A. Zhu Y. Yan L. et al. (2024). Human tactile sensing and sensorimotor mechanism: From afferent tactile signals to efferent motor control. *Nat. Commun.* 15:6857. 10.1038/s41467-024-50616-2 39127772 PMC11316806

[B35] World Medical Association (2013). *Declaration of Helsinki: Ethical Principles for Medical Research Involving Human Subjects.* France: World Medical Association. 10.1001/jama.2013.281053 24141714

[B36] Wu Q. Chan J. S. Yan J. H. (2016). Mild cognitive impairment affects motor control and skill learning. *Rev. Neurosci.* 27 197–217. 10.1515/revneuro-2015-0020 26426886

[B37] Xu G. Zhou M. Chen Y. Song Q. Sun W. Wang J. (2024). Brain activation during standing balance control in dual-task paradigm and its correlation among older adults with mild cognitive impairment: A fNIRS study. *BMC Geriatr.* 24:144. 10.1186/s12877-024-04772-1 38341561 PMC10859010

[B38] Xu J. Sun Y. Zhu X. Pan S. Tong Z. Jiang K. (2024). Tactile discrimination as a diagnostic indicator of cognitive decline in patients with mild cognitive impairment: A narrative review. *Heliyon* 10:e31256. 10.1016/j.heliyon.2024.e31256 38803967 PMC11129005

[B39] Yang J. Ogasa T. Ohta Y. Abe K. Wu J. (2010). Decline of human tactile angle discrimination in patients with mild cognitive impairment and Alzheimer’s disease. *J. Alzheimer’s Dis.* 22 225–234. 10.3233/jad-2010-100723 20847416

[B40] Zhang Z. Chen G. Zhang J. Yan T. Go R. Fukuyama H. et al. (2020). Tactile angle discrimination decreases due to subjective cognitive decline in Alzheimer’s disease. *Curr. Alzheimer Res.* 17 168–176. 10.2174/1567205017666200309104033 32148194

