# Correction: Tactile sensation moderates the association between hand dexterity and higher-level cognition in older adults with and without MCI

**DOI:** 10.3389/fnagi.2026.1927680

**Published:** 2026-07-20

**Authors:** Kimi Estela Kobayashi-Cuya, Ryota Sakurai, Susumu Ogawa, Hiroko Matsunaga, Keigo Hinakura, Shun Yoshikoshi, Yan Li, Maki Nishinakagawa, Hiroyuki Suzuki

**Affiliations:** 1 Research Team for Social Participation and Healthy Aging, Tokyo Metropolitan Institute for Geriatrics and Gerontology, Itabashi-ku, Tokyo, Japan; 2 Institute of Health and Sport Sciences, University of Tsukuba, Ibaraki, Japan; 3 Department of Nutrition and Dietetics, College of Nutrition, Kanto Gakuin University, Kanagawa, Japan

**Keywords:** aging, executive functioning, functional assessment, hand sensorimotor function, mild cognitive impairment, motor-cognitive integration, processing speed

There was a mistake in the formatting of Figure 1 as published. The spacing between the two graph columns was too compressed, and the left-column panels were not aligned with the left frame while the right-column panels were not aligned with the right frame. In addition, the overall size of the panels in Figure 1 is small relative to the amount of information presented, which limits readability. The corrected Figure 1 image appears below.

**Figure 1:**
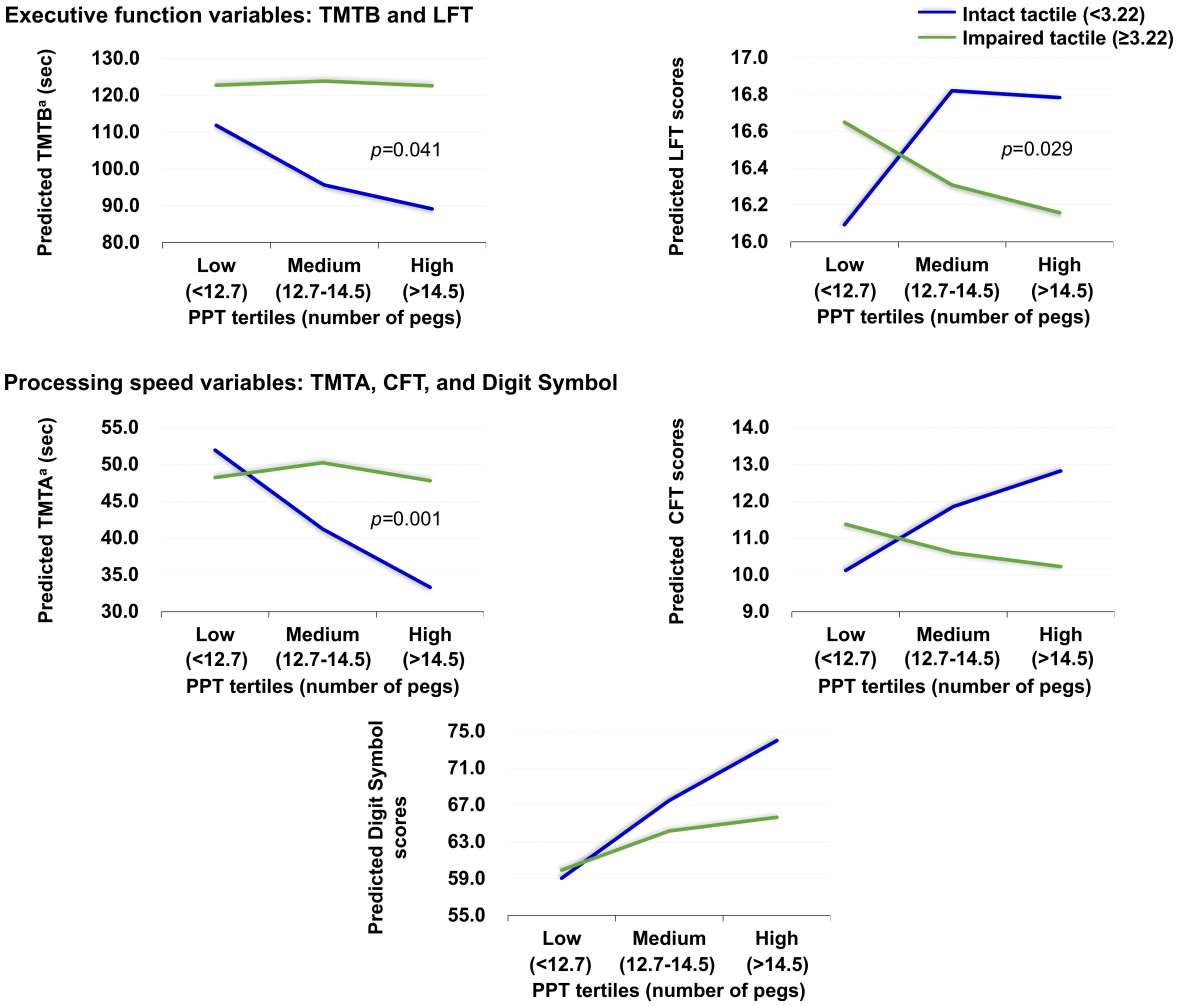
Moderation effects of tactile sensation on the association between hand dexterity and higher-level cognitive outcomes across PPT tertiles. Predicted scores for executive function (TMT-B, LFT) and processing speed (TMT-A, CFT, Digit Symbol) are plotted across PPT tertiles. Tactile sensation was stratified as intact (< 3.22) or impaired (≥3.22). PPT, Purdue Pegboard Test (number of pegs); TMT, Trail Making Test; LFT, Letter Fluency Test; CFT, Category Fluency Test. ^a^TMT-A and TMT-B values are back-transformed from the natural log scale to seconds for interpretability.

In the published article, the caption of Figure 1 and Figure 2 were erroneously swapped, with the caption intended for Figure 1 wrongly assigned to Figure 2, and the caption intended for Figure 2 wrongly assigned to Figure 1. The order has now been corrected; the corrected Figure captions appear below.

Figure 2. Conceptual model illustrating the moderating role of tactile sensation (*Z*) in the association between hand dexterity (*X*) and higher-level cognition (*Y*) in older adults. Significant *X* × *Z* interactions were observed for TMT-B, LFT, and TMT-A.

The original version of this article has been updated.

