# Supplementary material for: Tactile sensation moderates the association between hand dexterity and higher-level cognition in older adults with and without MCI
Source: Front Aging Neurosci. 2026 Jul 3;18:1833368. doi: 10.3389/fnagi.2026.1833368 (PMC13375975; doi:10.3389/fnagi.2026.1833368)
Supplement: Supplementary file 1 [file Table_1.docx]

**Supplementary Table S1.** Three-way interaction effects of tactile sensation and cognitive group on the association between hand dexterity and higher-level cognitive outcomes

| **Outcome** | **Dexterity (PPT)^a^  β(95%CI)** | **Tactile  β(95%CI)** | **Cog. group  β(95%CI)** | **Interaction  β(95%CI)** | **Interaction (p-value)** |
| --- | --- | --- | --- | --- | --- |
| **Executive function** | | | | | |
| TMT-B^b^ | 8.59  (-3.02,21.35) | -15.08  (-34.70,130.11) | -18.34  (-42.25,13.35) | 12.42  (-11.14,41.43) | 0.321 |
| LFT | 0.73  (-0.36,1.82) | -0.54  (-2.57,1.49) | 1.59  (-1.12,4.30) | 1.34  (-0.60,3.28) | 0.174 |
| **Processing speed** | | | | | |
| TMT-A^b^ | 1.86  (-2.01,6.16) | -2.24  (-10.12,7.27) | 2.28  (-8.91,16.74) | -3.82  (-11.19,4.76) | 0.348 |
| CFT | -0.15  (-1.14,0.84) | 0.29  (-2.01,2.58) | 0.97  (-2.08,4.02) | 0.47  (-1.71,2.66) | 0.430 |
| Digit Symbol | 0.69  (-2.84,4.23) | -0.72  (-8.91,7.47) | 4.85  (-6.05,15.75) | 3.66  (-4.16,11.47) | 0.355 |
| *Note.* PPT = Purdue Pegboard Test (number of pegs); TMT = Trail Making Test; LFT = Letter Fluency Test; CFT = Category Fluency Test. Each model included main effects and interaction terms for dexterity × tactile sensation, dexterity × cognitive group, tactile sensation × cognitive group, and the three-way interaction, and was adjusted for age, sex, education, GDS, TMIG-IC, comorbidities, and data source. Tactile sensation was coded as intact vs. impaired.  ^a^ Dexterity was mean-centered prior to analysis to facilitate interpretation of interaction terms.  ^b^ Values are back-transformed from the natural log scale to seconds for interpretability. | | | | | |
